# Linezolid-Associated Neurologic Adverse Events in Patients with Multidrug-Resistant Tuberculosis, France

**DOI:** 10.3201/eid2608.191499

**Published:** 2020-08

**Authors:** Marie Jaspard, Nathalie Butel, Najoua El Helali, Dhiba Marigot-Outtandy, Helene Guillot, Gilles Peytavin, Nicolas Veziris, Bahram Bodaghi, Philippe Flandre, Gregoire Petitjean, Eric Caumes, Valerie Pourcher

**Affiliations:** Sorbonne Université, Paris, France (M. Jaspard, H. Guillot, N. Veziris, B. Bodaghi, E. Caumes, V. Pourcher);; Assistance Publique–Hôpitaux de Paris, Hôpitaux Universitaires Pitié-Salpêtrière Charles Foix, Paris (M. Jaspard, H. Guillot, E. Caumes, V. Pourcher);; Assistance Publique – Hôpitaux de Paris, Groupe Hospitalier Pitié-Salpêtrière, Paris (N. Butel, B. Bodaghi);; Groupe Hospitalier Paris Saint Joseph, Paris (N. El Helali, G. Petitjean);; Bligny Medical Center, Briis sous Forges, France (D. Marigot-Outtandy);; Assistance Publique–Hôpitaux de Paris, Hôpital Bichat-Claude Bernard, Paris (G. Peytavin);; Université Paris Diderot, Sorbonne Paris Cité, Paris (G. Peytavin);; Assistance Publique–Hôpitaux de Paris, Hôpitaux Universitaires de l’Est Parisien, Paris (N. Veziris);; Institut Pierre Louis Institute d’Épidémiologie et de Santé Publique, Paris (P. Flandre, E. Caumes, V. Pourcher)

**Keywords:** Tuberculosis, MDR TB, XDR TB, TB, France, linezolid, neuropathy, pharmacokinetics, tuberculosis and other mycobacteria, neurologic events

## Abstract

Linezolid is one of the most effective drugs for treating multidrug-resistant tuberculosis (MDR TB), but adverse effects remain problematic. We evaluated 57 MDR TB patients who had received >1 dose of linezolid during 2011–2016. Overall, patients received 600 mg/day of linezolid for a median of 13 months. In 33 (58%) patients, neurologic or ophthalmologic signs developed, and 18 (32%) had confirmed peripheral neuropathy, which for 78% was irreversible at 12 months after the end of TB treatment despite linezolid withdrawal. Among the 19 patients who underwent ophthalmologic evaluation, 14 patients had optic neuropathy that fully reversed for 2. A total of 16 (33%) of 49 patients had a linezolid trough concentration >2 mg/L, and among these, 14 (88%) experienced adverse effects. No significant association was found between trough concentration and neurologic toxicity. These findings suggest the need to closely monitor patients for neurologic signs and discuss optimal duration of linezolid treatment.

Worldwide, tuberculosis (TB) affects 10 million persons every year. Among them, multidrug-resistant TB (MDR TB) is diagnosed for 484,000 (*1*); and among MDR TB cases, 10% are caused by extensively drug-resistant (XDR) strains of *Mycobacterium tuberculosis*. Outcomes for patients in TB-endemic countries are very poor, particularly for those with XDR TB; cure rates are <20% (*2*). However, even in TB-endemic countries, individualized treatment adapted to drug susceptibility achieves cure rates of only 60% in patients with XDR TB (*3*,*4*). Similarly, a meta-analysis of 50 studies performed in 25 countries and including 12,030 patients receiving individualized treatment for MDR TB found a pooled success rate of 61% for combination therapy regimens containing linezolid (*5*). At Pitié-Salpêtrière Hospital in Paris, France, the survival rate for XDR TB patients for whom highly effective drugs (e.g., linezolid, bedaquiline, or both) were prescribed was 80% (*6*–*8*). Overall, an 80% cure rate can be achieved for patients with MDR TB treated with a drug regimen that includes linezolid (*9*–*12*).

The 2014 World Health Organization (WHO) guidelines promote using highly active drugs for MDR TB, especially that caused by XDR TB strains (*13*). In 2016 and 2018, these recommendations were updated, and linezolid, along with bedaquiline and levofloxacin/moxifloxacin, were upgraded to group A drugs that should be offered to all patients (*14*,*15*).

Although linezolid is highly effective, its long-term use in patients with MDR TB is impaired by its adverse effects. Myelosuppression occurs in ≈30% of patients, particularly those receiving high doses (>600 mg/d) (*9*,*12*), and neurotoxicity with peripheral neuropathy is experienced by 30% of patients after 2 to 4 months of receiving low doses (<600 mg/d) (*12*). Furthermore, linezolid-associated optic neuropathy appears after 5 to 10 months of treatment for 30% of patients (*11*,*16*). It is still debated whether linezolid trough concentrations >2 mg/L (*17*) or long-term exposure could predict the occurrence of adverse effects (*18*,*19*). We therefore evaluated the occurrence and management of neurologic and ophthalmologic adverse effects among MDR TB patients receiving a linezolid-based treatment regimen.

## Methods

We retrospectively studied all patients with confirmed MDR TB who had received >1 dose of linezolid during January 2011–December 2016 and had been followed up for >12 months after the end of treatment. All patients had been admitted to Pitié-Salpêtrière Hospital, Paris, France, and most were discharged to Bligny Sanatorium and then followed up as outpatients. The TB drug regimen was adapted for each patient according to the results of drug susceptibility testing and discussion with the French Consilium team (*20*) in keeping with recommendations from the WHO and the Haut Conseil de la Santé Publique (*14*,*21*).

To monitor adverse effects of treatment, physicians performed daily neurologic and ophthalmologic examinations of hospitalized patients and monthly examinations of outpatients. Neurologic adverse effects included motor and sensory deficits, tendon reflex abnormalities, cranial nerve abnormalities, and pallesthesia. Ophthalmologic adverse effects included visual acuity loss, visual color abnormalities, or scotoma. If peripheral neuropathy was suspected, electromyography (EMG) was performed, including nerve conduction velocity (NCV) testing, to assess motor and sensory injuries to confirm the diagnosis. If a patient reported visual impairment indicating optic neuropathy, an ophthalmologist performed a specific clinical examination including Snellen best-corrected visual acuity measurement, relative afferent pupillary defect assessment, slit lamp examination, funduscopic examination, and color vision (Farnsworth D-15 Hue Test) and visual fields (Humphrey 24–2 SITA [Swedish Interactive Threshold Algorithm] standard or Goldmann) assessments. Nerve fiber layer thickness was evaluated by using optical coherence tomography for optic nerve abnormalities. If indicated, electrophysiologic measurement of visual evoked potential was also performed by an ophthalmologist. If an adverse effect was identified, follow-up frequency was determined by the physician, depending on severity of the effect. Neurologic and ophthalmologic adverse effect severity was classified by using the Common Terminology Criteria for Adverse Events (CTCAE) criteria (*22*).

Therapeutic drug monitoring for linezolid was performed at least once at the beginning of treatment (within the first month) for all patients except a few hospitalized at the beginning of 2011 when this analysis was not yet available at Pitié-Salpêtrière Hospital. If adverse effects occurred or if the dosage of linezolid was modified, serum concentration of linezolid was measured by using accredited ultra high–performance liquid chromatography–electrospray ionization/high-resolution mass spectrometry (European Medicines Agency, https://www.ema.europa.eu) and using stable isotope-labeled linezolid as an internal standard and a calibration range of 0.01–10.00 mg/L. We were able to follow patients for 12 months after end of treatment. Hence, we defined irreversibility of signs/symptoms as the persistence of signs/symptoms 12 months after the end of treatment.

We collected epidemiologic, clinical, and biological information from patients’ medical files. We investigated whether some variables were associated with neurologic toxicities by using a logistic regression model. Linezolid trough concentration was considered high if it was >2 mg/L at least once at baseline or during treatment. The data fulfilled the confidentiality criteria of the French National Data Protection “Commission Nationale de l'Informatique et des Libertés” (CNIL 2085894).

## Results

### Patient Characteristics

**Figure 1:**
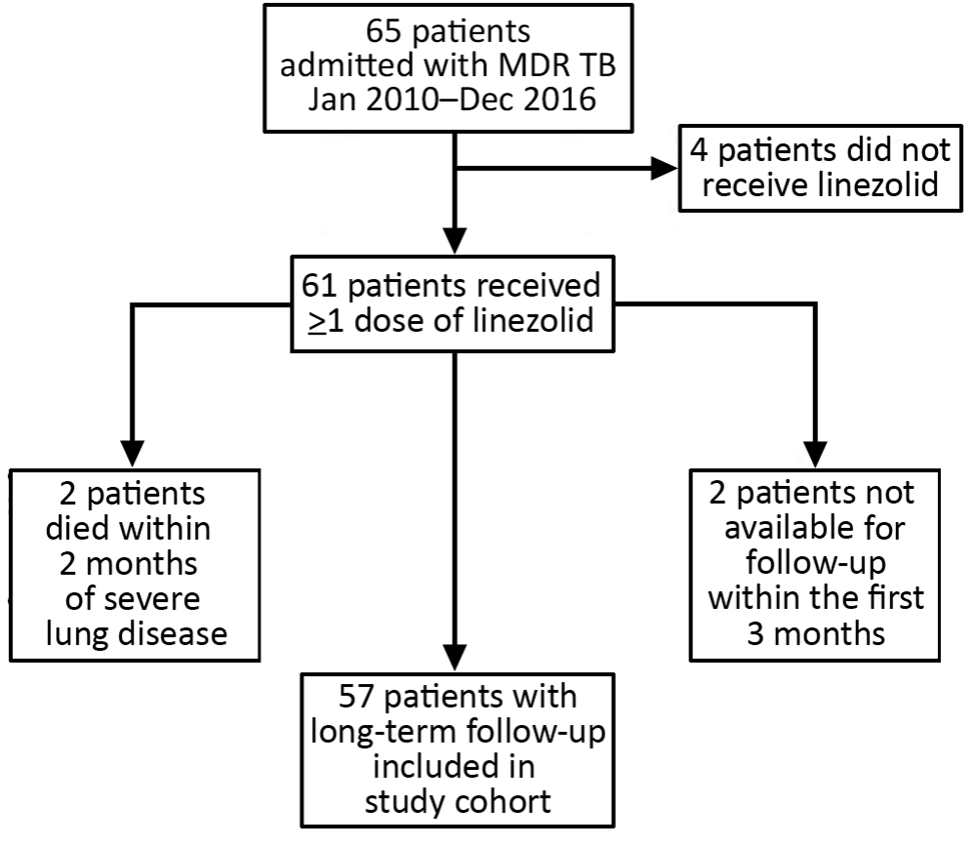
Outcomes for 65 patients with multidrug-resistant tuberculosis (MDR TB) admitted to Pitié-Salpêtrière Hospital, Paris, France, and included in study of linezolid-associated neurologic adverse events.

During January 2011–December 2016, MDR TB was diagnosed for 65 patients at Pitié-Salpêtrière Hospital. Four patients did not receive linezolid, 2 with severe pulmonary TB died within 2 months after initiation of treatment, and 2 were unavailable for follow-up within the first 3 months. We included in our study the remaining 57 patients for whom at least 12 months of follow-up information after the end of TB treatment was available (Figure 1; Tables 1, 2). 

**Table 1:** Sociodemographic and medical characteristics of 57 patients who received linezolid for multidrug-resistant tuberculosis, Pitié-Salpêtrière Hospital, Paris, France, 2011–2016*

Characteristic	Value
Age, y, median (IQR)	35 (26–39)
Sex	
M	39 (68)
F	18 (32)
Country or region of origin	
Georgia	36 (63)
Other Eastern Europe and Russia	8 (14)
Africa†	5 (9)
Asia‡	5 (9)
France	3 (5)
History of treated TB	37 (65)
History of isoniazid-based regimen	23 (40)
History of linezolid-based regimen	0
HIV infection	5 (9)
Hepatitis B surface antigen–positive	1 (2)
Hepatitis C	24 (42)
Diabetes mellitus	2 (4)
History of intravenous drug use	20 (35)
Opioid substitution therapy, methadone or buprenorphine	12 (21)
Alcohol consumption	8 (14)
Body mass index at first visit, median (IQR), kg/m^2^	20.1 (17.9–22.4)
Albumin level at first visit, median (IQR), g/L	34 (30–38)

*Values are no. (%) unless otherwise indicated. IQR, interquartile range; TB, tuberculosis.  †One from Algeria, 1 from Cameroon, 1 from Democratic Republic of the Congo, 2 from Côte d’Ivoire. ‡Three from China, 2 from Tibet.

**Table 2:** Tuberculosis drugs prescribed for >1 mo included in linezolid-based regimen for 57 patients with multidrug-resistant tuberculosis, Pitié-Salpêtrière Hospital, Paris, France, 2011–2016*

Drug	No. (%) patients
Cycloserine	49 (86)
Para-aminosalicylic acid	49 (86)
Amikacin	41 (72)
Bedaquiline	40 (70)
Pyrazinamide	37 (65)
Moxifloxacin	33 (58)
Ethambutol	17 (30)
Ethionamide	13 (23)
Levofloxacin	6 (11)
Capreomycin	3 (5)
Delamanid	3 (5)
Meropenem/clavulanate	2 (4)

Among the 57 patients, 11 (19%) were infected with an MDR strain of *M. tuberculosis*, 20 (35%) with a pre-XDR strain (resistance to isoniazid and rifampicin and either a fluoroquinolone or a second-line injectable agent but not both), and 26 (46%) with an XDR strain. A total of 55 patients had pulmonary TB, 2 had extrapulmonary TB (bone), and 11 had both (bone, lymph node, genital, or laryngeal). Of the 55 with pulmonary TB, the lesions involved both lungs for 42 (76%) patients, and cavitation was found in 39 (71%) patients.

Median duration of TB treatment was 19.4 months (interquartile range [IQR] 17.3–24.0 months). The linezolid dose was 600 mg once daily for all patients; no patient received >600 mg/day. The median duration of linezolid treatment was 12.8 months (IQR 6.0– 19.2 months).

Among the 57 patients, treatment was successful, according to the WHO outcome definition (*23*), for 52 (91%); 3 were unavailable for follow-up, and 2 died with severe neurologic signs suggestive of serotonin syndrome. However, we cannot formally link these deaths to linezolid because of doubts about the degree of attribution. 

One of the patients who died had arrived in France from Georgia in 2012 with XDR TB, hepatitis C virus co-infection, and a history of diabetes mellitus and high blood pressure. While in Georgia, he had received treatment for relapsing MDR TB for several years and received several drugs (para-aminosalicylic acid [PAS], cycloserine, ethionamide, moxifloxacin, clofazimine, clarithromycin, and capreomycin). When he arrived at the infectious disease department at Pitié Salpêtrière Hospital, and based on the French Consilium recommendations for patients with XDR TB, we initiated treatment with cycloserine, PAS, bedaquiline, meropenem/clavulanate, and linezolid. Treatment for diabetes (insulin) and high blood pressure (perindopril) was also started. Eleven months later, peripheral neuropathy appeared (confirmed by EMG), as well as orthostatic hypotension and mental confusion. We were reticent to change the TB treatment because of the highly resistant strain and severe lung disease (right upper lung lobectomy was performed after 10 months of TB treatment). Fifteen months after starting TB treatment, the patient suddenly experienced dysphagia, laryngeal hypoesthesia, dysarthria, bilateral myosis, myoclonus, hearing impairment, and visual impairment. Magnetic resonance imaging, electroencephalography, and cerebrospinal fluid analysis findings were unremarkable. TB treatment was immediately stopped, but the patient died; no cause was identified during autopsy. The patient’s linezolid concentration was 0.2 mg/L 1 month after treatment start and 0.6 mg/L on the last day of treatment. 

For the other patient who died, XDR TB was diagnosed and treatment began when he arrived in France from Armenia; initial treatment was PAS, bedaquiline, amikacin, meropenem/clavulanate, and linezolid, which was stopped rapidly because of poor tolerance. Previous TB treatment history in Armenia was unavailable. He also underwent a left upper lung lobectomy because of the absence of sputum culture conversion; was co-infected with the hepatitis C virus; and was addicted to tobacco, alcohol, and intravenous drugs. Five months after TB treatment was initiated, peripheral neuropathy developed (confirmed by EMG), although trough linezolid concentration was only 0.6 mg/L. During month 7 of treatment, trough linezolid concentration increased to 2.6 mg/L but otherwise remained <2 mg/L. After 12 months of TB treatment, the patient experienced a psychological disorder; sequential treatment consisted of loxapine, amitriptyline, mianserin, or oxazepam. Peripheral neuropathy worsened, causing severe leg pain requiring analgesia (opioids, pregabalin, clonazepam, gabapentin). At 15 months, linezolid was discontinued, and at 18 months, the remaining TB treatment was discontinued. A few days later, the patient was transferred to intensive care with dysphagia, loss of cough and tendon reflexes, distended bladder, sensory neuropathy, motor deficits, and pneumonia. All these signs and symptoms suggested brainstem injury or diffuse neuropathy. He died suddenly a few hours later. No autopsy was performed.

### Adverse Effects

**Figure 2:**
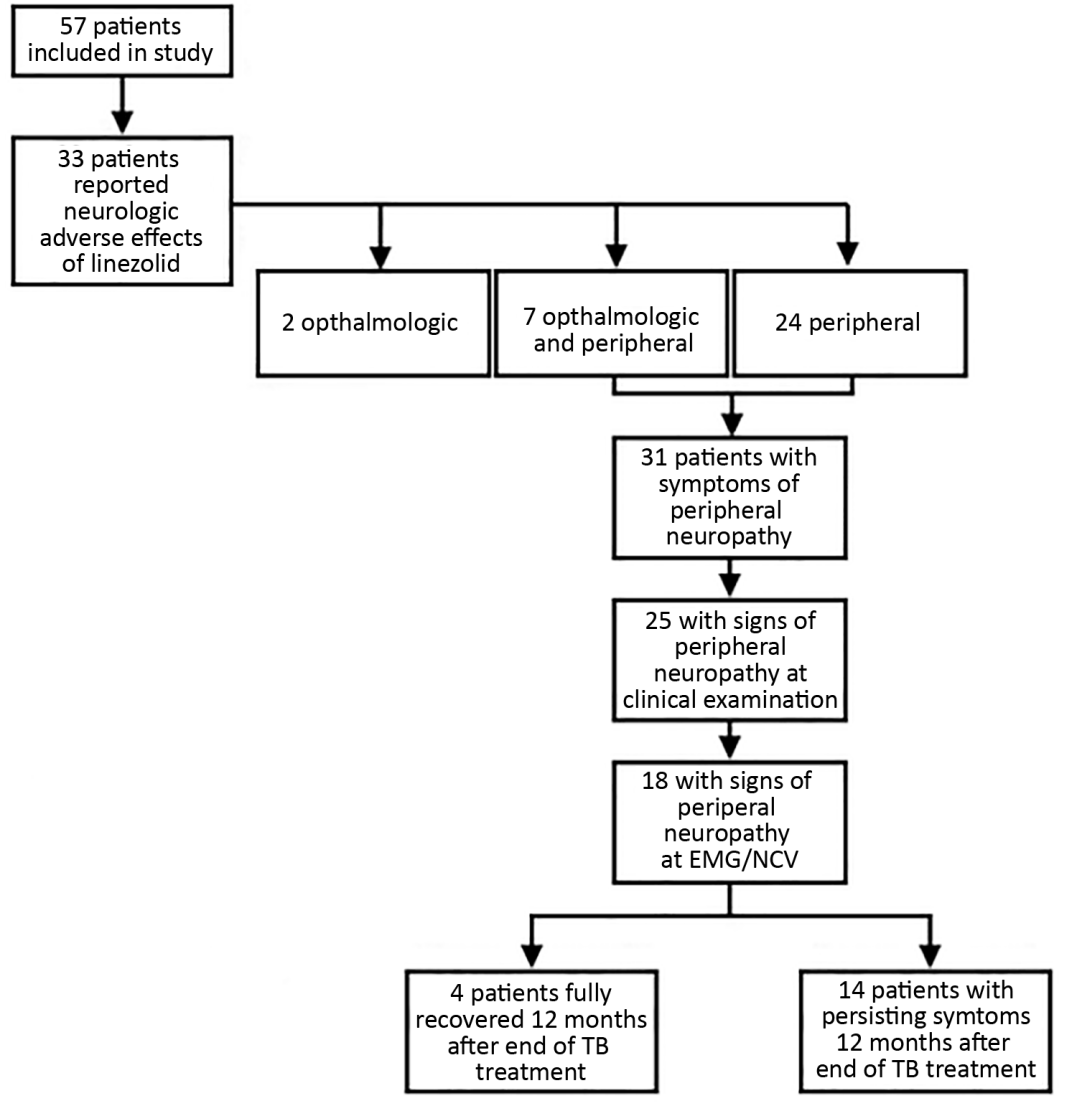
Peripheral neuropathy occurrence and evolution among the 57 patients who received linezolid for multidrug-resistant tuberculosis during 2011 and 2016, France. EMG, electromyelogram; NCV, nerve-conduction velocity testing; TB, tuberculosis.

Overall, 33 (58%) patients reported ophthalmologic or neurologic signs/symptoms, possibly linked to linezolid exposure: 24 patients had peripheral neuropathologic signs/symptoms only, 2 patients had ophthalmologic signs/symptoms only, and 7 patients had both (Figure 2). Moreover, linezolid was stopped because of neurologic adverse effects for 23 patients (18 for peripheral neuropathy, 1 for optical neuropathy, and 4 for both neuropathies) after a median duration of linezolid exposure of 8.6 months (IQR 5.6–15.3 months).

Among the 57 patients, 31 reported peripheral neurologic signs/symptoms (24 peripheral only and 7 both peripheral and optical), peripheral sensory or motor neuropathy signs were detected during clinical examination for 25, and neuropathy was confirmed by EMG or NCV for 18 (32%) of the 57 (Figure 2). Among the 31 with neuropathy signs/symptoms, 12 were CTCAE classification grade 1, 18 were grade 2, and 1 was grade 4. Among the 18 patients with peripheral neuropathy confirmed by EMG/NCV, 14 (78%) had persistent signs/symptoms for at least 12 months after discontinuation of TB treatment. However, language barriers and precarious situations faced by these patients made evaluating sequelae severity difficult. Although all patients were ambulatory, 16 required long-term (until the end of the 12 months of follow-up) analgesic therapy such as pregabalin for 12 (75%) and paracetamol, levetiracetam, or opioids for the others.

Ophthalmologic testing was performed for 19 patients, of which 13 were routinely tested at the discretion of the treating physician, for fear of sides effects resulting from long-term exposure to linezolid, and 6 were tested after reporting optical signs/symptoms. Of the 19 patients tested, 14 (9 without complaints and 5 with complaints) had optic neuropathy (Table 3) attributed to linezolid after exclusion of other etiologies. Among these patients, CTCAE severity was grade 1 for 10 patients, grade 2 for 2 patients, and grade 4 for 2 patients.

**Table 3:** Characteristics of 14 patients who experienced optic neuropathy after linezolid treatment for multidrug-resistant tuberculosis, Pitié-Salpêtrière Hospital, Paris, France, 2011–2016*

Patient age, y/sex,	Fundus examination	Visual color†	Visual field	Involvement	Other	Linezolid duration, mo	Months from start of linezolid to onset of optic neuropathy	Reversibility after linezolid withdrawal
37/M	Papillary edema	Tritanopia	BSE	B	None	3.4	0.0	No
42/M	WNL	Tritanopia	Aciform scotoma	B	None	13.5	1.4	No
20/M	WNL	Tritanopia	WNL	B	None	18.5	1.6	Yes
40/M	WNL	Tritanopia	BSE	B	Optic neuropathy confirmed by VEP	15.3	14.6	Yes
48/M	WNL	Tritan	BSE	B	None	5.7	9.3	No
43/M	WNL	Tritanopia	NA	U	None	11.6	17.9	Yes
40/M	WNL	Tritanopia	BSE	U	None	18	NA	Yes
39/M	WNL	Tritanopia	BSE	B	None	23.1	15.4	Yes
42/M	WNL	Tritanopia	BSE	B	None	19.4	5.2	Yes
40/M	WNL	Tritanopia	NA	B		4.4	1.0	No
34/M	Papillary edema	Tritanopia	BSE	B	Optic neuropathy confirmed by VEP	16.6	15.1	Yes
21/M	Papillary edema	Tritanopia	Scotoma	B	Optic neuropathy confirmed by VEP	7.1	8.2	Yes
23/F	Hyperemia of optical nerve	Tritanopia	BSE	B	None	NA	10.2	Yes
30/M	Hyperemia of optical nerve	Tritan	BSE	B	None	19.1	16.2	Yes

*B, bilateral; BSE, blind spot enlargement; NA, not available; U, unilateral, VEP, visual evoked potential; WNL, within normal limits. †Tritan, common blue–yellow color blindness.; tritarnopia, blue–yellow confusion typical in patients with optic neuropathies.

After linezolid withdrawal, a second ophthalmologic examination for 9 patients indicated that 2 had fully recovered, 3 had improved visual acuity with residual impaired vision, and 4 showed no improvement. Five patients did not undergo a second examination, possibly because they had clinical improvement and did not report optical signs/symptoms.

The median delay between linezolid initiation and occurrence of neurologic adverse effects was 8.3 (IQR 4–11) months and of ophthalmologic adverse effects was 9.3 (IQR 1.6 –15.1) months. Overall, 27 (47%) of the 57 patients had neurologic side effects confirmed by EMG/NCV or specific ophthalmologic examination, 13 (23%) had peripheral neuropathy only, 9 (16%) had ophthalmologic neuropathy only, and 5 (9%) had both. Linezolid treatment duration was 15.1 (IQR 7.2–19.1) months for patients with confirmed neurologic adverse effects and 12.3 (IQR 5.5–21.1) months for those without (p = 0.9148).

### Linezolid Trough Concentrations and Neuropathy

Linezolid trough concentrations were available for 49 patients and were analyzed in association with neuropathy (Table 4). Among the 16 (33%) patients with trough concentrations >2 mg/L (median 3 [IQR 2.4–3.7] mg/L) at least once at baseline or during treatment, 14 (88%) experienced linezolid-associated adverse effects: 9 had a peripheral neuropathy and 5 had optical neuropathy. Among the 33 (67%) patients with all trough concentrations <2 mg/L (median 0.4 [IQR 0.19–1] mg/L), 27 (82%) experienced linezolid-associated adverse effects: 17 had peripheral neuropathy and 10 had optical neuropathy. Among the 8 patients for whom linezolid concentration was not evaluated, linezolid-associated adverse effects developed in 6 (75%) patients: 5 had peripheral neuropathy and 1 had optic neuropathy. We found no association between development of neuropathy and demographic characteristics, trough concentration, or linezolid treatment duration (p>0.05) (Table 5).

**Table 4:** Neuropathy according to linezolid trough concentration among of 57 patients with multidrug-resistant tuberculosis, Pitié-Salpêtrière Hospital, Paris, France, 2011–2016

Neuropathy	No. patients	Linezolid trough concentration		
		>2 mg/L, n = 16	≤2 mg/L, n = 33	Not available, n = 8
Clinical peripheral	31	9	17	5
Confirmed peripheral	18	5	10	3
Clinical optical	9	5	3	1
Confirmed optical	14	4	10	0
Peripheral + optical	10	3	6	1

**Table 5:** Association between clinical and confirmed peripheral and optical neuropathy and patient characteristics among 57 patients with multidrug-resistant tuberculosis, Pitié-Salpêtrière Hospital, Paris, France, 2011–2016*

Characteristic	Clinical neuropathy			Confirmed neuropathy	
	Value	p value*		Value	p value*
Age, OR (95% CI)†	1.83 (0.9–3.7)	0.09		1.66 (0.8–3.3)	0.14
Immunosuppression treatment, OR (95% CI)	0.91 (0.3–2.7)	0.98		2.1 (0.7–6.2)	0.18
History of intravenous drug use, OR (95% CI)	0.43 (0.5–4.9)	0.43		1.79 (0.6–5.4)	0.30
Opioid substitution therapy, OR (95% CI)	1.6 (0.4–6.1)	0.49		1.92 (0.5–7)	0.32
Albumin, g/L	0.81 (0.4–1.8)	0.61		1.01 (0.5–2.2)	0.98
Linezolid treatment duration, mo	0.47 (0.2–1.2)	0.10		1.11 (0.5–2.7)	0.82
Culture conversion, d	0.93 (0.8–1.1)	0.35		1.15 (0.9–1.4)	0.21
Trough linezolid concentration >2 mg/L	2.07 (0.6–7.3)	0.26		0.83 (0.3–2.7)	0.76

*p values from univariate logistic models. OR, odds ratio. †Risk based on age increase by 10-y intervals.

## Discussion

In our study, 58% of MDR/XDR TB patients reported a neurologic or ophthalmologic disorder possibly associated with linezolid exposure; neuropathies were confirmed for 47%. Linezolid-related peripheral neuropathy was confirmed (by EMG/NCV) for almost 32%. Neuropathy and demographic characteristics did not seem to be associated with trough concentration, or linezolid duration.

Linezolid-related optic neuropathies in patients receiving treatment for *Staphylococcus aureus* infections have been described (*24*) and can be asymptomatic or lead to reduced visual acuity, blurred vision, central scotoma, and dyschromatopsia (*19*,*25*). We found a high prevalence of confirmed optic neuropathy (25% of our cohort) that was fully reversible (meaning complete recovery of ophthalmologic disorder 12 months after the end of TB treatment) for only 2 of our patients after treatment withdrawal. We therefore suggest testing visual acuity and performing a sensory color vision test and a Goldmann visual field test before initiating linezolid with monthly checkups, as has been recently recommended (*19*).

Our finding of a peripheral neuropathy rate of 32% is similar to the neuropathy rate of 30% reported in a recent meta-analysis (*12*). In our study, peripheral neuropathy diagnosis was confirmed after a median duration of 8 months of treatment. In contrast, a study in China found that peripheral neuropathy occurred 2 to 4 months after treatment start (*11*). This difference can be explained by the high daily doses of linezolid received by patients in that study (1,200 mg/d loading dose for 6 weeks, then 600 mg/d) compared with the 600 mg/day received by all patients in our study. Various studies report irreversible neuropathy (*10*,*12*,*26**,**27*). In our study, 78% of patients with confirmed peripheral neuropathy did not fully recover despite linezolid withdrawal. Those patients were followed up for 12 months after the end of their TB treatment. Some may have recovered after that follow-up period. Thus, this result should encourage closer monitoring of neurologic complaints with detailed and regular clinical neurologic examinations. Linezolid should also be immediately discontinued at the onset of neurologic signs/symptoms.

Questions persist about the 2 patients who died. First, both patients had concurrent conditions known to increase the risk for neurologic disorders. One patient had hepatitis C, diabetes, and high blood pressure; the other had hepatitis C and a history of substance abuse. Second, linezolid treatment was continued for both patients despite development of neurologic signs/symptoms. At that time, in 2013, TB treatment was continued in the context of a highly resistant strain of *M. tuberculosis*, lack of an effective drug (e.g., delamanid) for treatment, and severe disease, despite daily in-patient monitoring. In hindsight, stopping any neurotoxic treatments as soon as neurologic signs/symptoms occurred may have been wise. Third, the second patient received several psychotropic drugs, which can potentiate the linezolid effect and induce serotonin syndrome. Linezolid-associated serotonin syndrome has been described in association with use of various drugs such as metoclopramide, paroxetine, or amitriptyline (*28**,**29*). Hence, when long-term use of linezolid is unavoidable because of highly resistant TB, drugs that induce serotonin syndrome must not be prescribed.

Another way to monitor adverse effects of antimicrobial drugs is to systematically measure serum trough concentrations. The role of linezolid dosage, treatment duration, and cumulative dose on neuropathy occurrence is still debated. Bolhuis et al. stated that the linezolid area under the curve over 24 hours does not affect adverse effect occurrence but that long exposure duration and cumulative doses do (*18*). Song et al. demonstrated a direct correlation between linezolid dose, trough concentrations, and development of clinical toxicity. They found that adverse effects developed in all patients for whom mean linezolid trough was >2 mg/L, whereas they developed in >50% of those for whom mean linezolid trough was <2 mg/L (*17*). In our study, linezolid trough concentration was not associated with development of neurologic side effects. The fact that our study was retrospective could explain why our findings were not consistent with those of Song et al. (*17*).

When we compared patients with and without clinical or confirmed peripheral or optical neuropathy, we found no demographic characteristics associated with neuropathy occurrence (Table 5). However, in a study of MDR TB patients in India, the high rate of neuropathy was explained by a higher proportion of malnourished patients (*27*). For specific TB parameters, we found no association between the onset of neurologic adverse effects and trough concentration or linezolid treatment duration. Neuropathy seems to be independent of linezolid trough concentration (88% for >2 mg/L, 82% for <2 mg/L, 75% for those without measurement) or linezolid treatment duration. In addition, linezolid treatment duration was shorter among patients who did not experience neurologic adverse effects than among patients who did, but this finding was not statistically significant, probably because of small sample size. 

Although the 2018 WHO recommendations state that linezolid should be offered to all patients, the optimal treatment duration has not yet been established (*15*). The high prevalence of linezolid toxicity in our cohort, together with the lack of reversibility for more than half of the patients, is not in favor of long linezolid treatment durations for MDR TB patients. Furthermore, recent findings regarding the short duration of treatment regimens for MDR TB (*30*–*32*) have led the WHO to recommend 9–12 months of therapy if particular patient criteria apply (*33*). Hence, because of the high level of linezolid neurotoxicity and a general trend to decrease MDR TB treatment duration, we suggest that exposure to linezolid should be as short as possible to avoid development of irreversible side effects (*34*). Close monitoring should also be offered to all patients receiving linezolid. In our study, 72% of patients with signs of neuropathy at clinical examination were found to have EMG/NCV abnormalities. Using clinical examination to screen for peripheral neuropathy is therefore effective, even if EMG/NCV is unavailable. Ophthalmologic examination is more complicated. Ophthalmologic abnormalities were found in 9 of 13 patients without signs/symptoms and in 5 of 6 patients with signs/symptoms. We are unable to make clear recommendations regarding ophthalmologic testing for this patient population. However, we felt it necessary to raise awareness about linezolid toxicity and highlight the need to evaluate visual acuity as often as possible for patients receiving long-term linezolid.

Our monocentric study is limited by its retrospective design. The data were incomplete for several key variables, such as pharmacokinetics and pharmacodynamics, which could explain the difference between our findings and those of the study by Song et al. (*17*). The overall sample size in each patient group was small and may have been insufficiently powered to show differences between groups. The fact that 42% (24/57) of patients had hepatitis C and 14% (8/57) had alcohol dependency might increase the risk for peripheral neuropathy, independent of linezolid exposure. Moreover, among the 17 patients who received concomitant ethambutol, 8 had confirmed optic neuropathy. Although the ophthalmologic injuries resulting from linezolid differ from those resulting from ethambutol, completely excluding ethambutol toxicity in our findings is difficult. Thus, whenever possible, ethambutol use in a linezolid-based regimen must be avoided. Last, clinical examinations regarding ophthalmologic adverse effects were performed according to physician discretion without a systematic algorithm for recording adverse effects, which can bias the exact number of patients affected. This limitation, linked to the retrospective nature of the study, raises the issue of systematic ophthalmologic monitoring for patients receiving linezolid. One minor limitation is that only patients followed up for 12 months were included in the study. Two patients stopped coming to follow-up visits, possibly because of adverse linezolid events; thus, adverse events may have been underreported. However, these patients left during the first 3 months, probably before any neurologic adverse effects had developed. 

Our study does, however, reflect the real difficulties of medical care and treatment surveillance of patients with highly resistant TB and serves to warn clinicians about long-term linezolid adverse effects. It also raises the crucial question of linezolid treatment duration for MDR TB patients. The patients in our study were in precarious economic and social situations; language barriers might explain the high rate of neurologic complications because patients might not have been able to explain their symptoms soon enough or might not have been sufficiently aware of the potential adverse effects of linezolid despite detailed explanations delivered by translators (by phone or internet). Overall, management was challenging for those patients, a situation that may occur elsewhere.

In conclusion, our study illustrates that MDR TB patients receiving linezolid should be monitored because neurologic adverse effects are severe, frequent, and often irreversible (at least at 12 months after treatment termination). Further studies are needed to evaluate the risk factors associated with linezolid toxicity and to evaluate the best treatment duration to decrease the rate of neurologic adverse effects without affecting MDR TB outcomes. However, systematic clinical examination should be implemented for all patients before treatment and monthly thereafter so that linezolid withdrawal can be discussed if neuropathy develops. 

## References

[1] World Health Organization. Global tuberculosis report 2018 [cited 2020 Jun 10]. https://apps.who.int/iris/handle/10665/274453

[2] Kim H-R, Hwang SS, Kim HJ, Lee SM, Yoo C-G, Kim YW, et al. Impact of extensive drug resistance on treatment outcomes in non-HIV-infected patients with multidrug-resistant tuberculosis. Clin Infect Dis. 2007;45:1290–5. 10.1086/522537 17968823

[3] Velásquez GE, Becerra MC, Gelmanova IY, Pasechnikov AD, Yedilbayev A, Shin SS, et al. Improving outcomes for multidrug-resistant tuberculosis: aggressive regimens prevent treatment failure and death. Clin Infect Dis. 2014;59:9–15. 10.1093/cid/ciu209 24729493 PMC4305127

[4] Mitnick CD, Shin SS, Seung KJ, Rich ML, Atwood SS, Furin JJ, et al. Comprehensive treatment of extensively drug-resistant tuberculosis. N Engl J Med. 2008;359:563–74. 10.1056/NEJMoa0800106 18687637 PMC2673722

[5] Collaborative Group for the Meta-Analysis of Individual Patient Data in MDR-TB Treatment–2017; Ahmad N, Ahuja SD, Akkerman OW, Alffenaar J-WC, Anderson LF, Baghaei P, et al. Treatment correlates of successful outcomes in pulmonary multidrug-resistant tuberculosis: an individual patient data meta-analysis. Lancet. 2018 392:821–34. 10.1016/S0140-6736(18)31644-1 PMC6463280 30215381

[6] Guglielmetti L, Jaspard M, Le Dû D, Lachâtre M, Marigot-Outtandy D, Bernard C, et al. Long-term outcome and safety of prolonged bedaquiline treatment for multidrug-resistant tuberculosis. Eur Respir J. 2016. 10.1183/13993003.00411-2016 28182570

[7] Henry B, Revest M, Dournon N, Epelboin L, Mellon G, Bellaud G, et al. Preliminary favorable outcome for medically and surgically managed extensively drug-resistant tuberculosis, France, 2009-2014. Emerg Infect Dis. 2016;22:518–21. 10.3201/eid2203.151130 26891089 PMC4766883

[8] Guglielmetti L, Le Dû D, Jachym M, Henry B, Martin D, Caumes E, et al.; MDR-TB Management Group of the French National Reference Center for Mycobacteria and the Physicians of the French MDR-TB Cohort. Compassionate use of bedaquiline for the treatment of multidrug-resistant and extensively drug-resistant tuberculosis: interim analysis of a French cohort. Clin Infect Dis. 2015;60:188–94. 10.1093/cid/ciu786 25320286

[9] Zhang X, Falagas ME, Vardakas KZ, Wang R, Qin R, Wang J, et al. Systematic review and meta-analysis of the efficacy and safety of therapy with linezolid containing regimens in the treatment of multidrug-resistant and extensively drug-resistant tuberculosis. J Thorac Dis. 2015;7:603–15. 25973226 10.3978/j.issn.2072-1439.2015.03.10 PMC4419320

[10] Lee M, Lee J, Carroll MW, Choi H, Min S, Song T, et al. Linezolid for treatment of chronic extensively drug-resistant tuberculosis. N Engl J Med. 2012;367:1508–18. 10.1056/NEJMoa1201964 23075177 PMC3814175

[11] Tang S, Yao L, Hao X, Zhang X, Liu G, Liu X, et al. Efficacy, safety and tolerability of linezolid for the treatment of XDR-TB: a study in China. Eur Respir J. 2015;45:161–70. 10.1183/09031936.00035114 25234807

[12] Agyeman AA, Ofori-Asenso R. Efficacy and safety profile of linezolid in the treatment of multidrug-resistant (MDR) and extensively drug-resistant (XDR) tuberculosis: a systematic review and meta-analysis. Ann Clin Microbiol Antimicrob. 2016;15:41. 10.1186/s12941-016-0156-y 27334498 PMC4917997

[13] World Health Organization. Companion handbook to the WHO guidelines for the programmatic management of drug-resistant tuberculosis [cited 2020 Jun 10]. http://www.who.int/tb/publications/pmdt_companionhandbook 25320836

[14] World Health Organization. WHO treatment guidelines for drug-resistant tuberculosis; 2016 update [cited 2017 Jul 10]. http://www.who.int/tb/areas-of-work/drug-resistant-tb/MDRTBguidelines2016.pdf

[15] World Health Organization. Rapid communication: key changes to treatment of multidrug- and rifampicin-resistant tuberculosis (MDR/RR-TB) [cited 2020 Jun 10]. http://www.who.int/tb/publications/2018/rapid_communications_MDR

[16] Mehta S, Das M, Laxmeshwar C, Jonckheere S, Thi SS, Isaakidis P. Linezolid-associated optic neuropathy in drug-resistant tuberculosis patients in Mumbai, India. PLoS One. 2016;11:e0162138. 10.1371/journal.pone.0162138 27611434 PMC5017632

[17] Song T, Lee M, Jeon H-S, Park Y, Dodd LE, Dartois V, et al. Linezolid trough concentrations correlate with mitochondrial toxicity-related adverse events in the treatment of chronic extensively drug-resistant tuberculosis. EBioMedicine. 2015;2:1627–33. 10.1016/j.ebiom.2015.09.051 26870788 PMC4740314

[18] Bolhuis MS, Tiberi S, Sotgiu G, De Lorenzo S, Kosterink JGW, van der Werf TS, et al. Linezolid tolerability in multidrug-resistant tuberculosis: a retrospective study. Eur Respir J. 2015;46:1205–7. 10.1183/13993003.00606-2015 26160870

[19] Dempsey SP, Sickman A, Slagle WS. Case report: linezolid optic neuropathy and proposed evidenced-based screening recommendation. Optom Vis Sci. 2018;95:468–74. 10.1097/OPX.0000000000001216 29683987

[20] Guglielmetti L, Jaffré J, Bernard C, Brossier F, El Helali N, Chadelat K, et al. Multidisciplinary advisory teams to manage multidrug-resistant tuberculosis: the example of the French Consilium. Int J Tuberc Lung Dis. 2019;23:1050–4. 10.5588/ijtld.18.0779 31627768

[21] Haut Conseil de la Santé Publique. Tuberculose à bacilles résistants: diagnostic et prise en charge. Lignes directices. 2014 [cited 2020 Jun 10]. https://www.hcsp.fr/explore.cgi/avisrapportsdomaine?clefr=483

[22] National Institutes of Health, National Cancer Institute. Common terminology criteria for adverse events (CTCAE) version 5.0 [cited 2020 Jun 10]. https://ctep.cancer.gov/protocolDevelopment/electronic_applications/ctc.htm

[23] Laserson KF, Thorpe LE, Leimane V, Weyer K, Mitnick CD, Riekstina V, et al. Speaking the same language: treatment outcome definitions for multidrug-resistant tuberculosis. Int J Tuberc Lung Dis. 2005;9:640–5. 15971391

[24] Lee E, Burger S, Shah J, Melton C, Mullen M, Warren F, et al. Linezolid-associated toxic optic neuropathy: a report of 2 cases. Clin Infect Dis. 2003;37:1389–91. 10.1086/379012 14583875

[25] Karuppannasamy D, Raghuram A, Sundar D. Linezolid-induced optic neuropathy. Indian J Ophthalmol. 2014;62:497–500. 10.4103/0301-4738.118451 24088636 PMC4064234

[26] Xu H-B, Jiang R-H, Li L, Xiao H-P. Linezolid in the treatment of MDR-TB: a retrospective clinical study. Int J Tuberc Lung Dis. 2012;16:358–63. 10.5588/ijtld.11.0493 22640450

[27] Udwadia ZF, Sen T, Moharil G. Assessment of linezolid efficacy and safety in MDR- and XDR-TB: an Indian perspective. Eur Respir J. 2010;35:936–8, author reply 938–40. 10.1183/09031936.00132009 20356997

[28] Lalondriz Bueno YM, Monereo Muñoz M-B, Ripper C-J, Santolaria F. [Serotoninergic syndrome due to linezolid and metoclopramide] [in Spanish]. Enferm Infecc Microbiol Clin. 2014;32:690–1. 10.1016/j.eimc.2014.03.013 24873980

[29] Morales-Molina JA, Mateu-de Antonio J, Grau Cerrato S, Marín Casino M. [Likely serotoninergic syndrome from an interaction between amitryptiline, paroxetine, and linezolid] [in Spanish]. Farm Hosp. 2005;29:292–3. 16268747

[30] Aung KJM, Van Deun A, Declercq E, Sarker MR, Das PK, Hossain MA, et al. Successful ‘9-month Bangladesh regimen’ for multidrug-resistant tuberculosis among over 500 consecutive patients. Int J Tuberc Lung Dis. 2014;18:1180–7. 10.5588/ijtld.14.0100 25216831

[31] Van Deun A, Maug AKJ, Salim MAH, Das PK, Sarker MR, Daru P, et al. Short, highly effective, and inexpensive standardized treatment of multidrug-resistant tuberculosis. Am J Respir Crit Care Med. 2010;182:684–92. 10.1164/rccm.201001-0077OC 20442432

[32] Nunn AJ, Phillips PPJ, Meredith SK, Chiang C-Y, Conradie F, Dalai D, et al.; STREAM Study Collaborators. A trial of a shorter regimen for rifampin-resistant tuberculosis. N Engl J Med. 2019;380:1201–13. 10.1056/NEJMoa1811867 30865791

[33] World Health Organization. Rapid communication: key changes to the treatment of drug-resistant tuberculosis. 2019 Dec [cited 2020 Jun 10]. http://www.who.int/tb/publications/2019/rapid_communications_MDR

[34] Conradie F, Diacon AH, Everitt D, Mendel C, Van Niekerk C, Howell P, et al. The NIX-TB trial of pretomanid, bedaquiline and linezolid to treat XDR-TB. Abstract 80-LB. Presented at: Conference on Retroviruses and Opportunistic Infections. 2017 Feb 13–16; Seattle, WA, USA.

